# Diffusion-Weighted Magnetic Resonance Imaging Is an Ideal Imaging Method to Detect Infection in Pancreatic Collections: A Brief Primer for the Gastroenterologists

**DOI:** 10.7759/cureus.21530

**Published:** 2022-01-23

**Authors:** Binit Sureka, Balwant Rai, Vaibhav K Varshney, Vijaya Lakshmi Nag, Mahendra Kumar Garg, Pawan Garg, Taruna Yadav, Pushpinder S Khera

**Affiliations:** 1 Diagnostic & Interventional Radiology, All India Institute of Medical Sciences, Jodhpur, Jodhpur, IND; 2 Surgical Gastroenterology, All India Institute of Medical Sciences, Jodhpur, Jodhpur, IND; 3 Microbiology, All India Institute of Medical Sciences, Jodhpur, Jodhpur, IND; 4 Medicine, All India Institute of Medical Sciences, Jodhpur, Jodhpur, IND

**Keywords:** sterile, pancreatic collections, infected, diffusion mri, ct

## Abstract

Background: The development of infection in pancreatitis significantly increases the mortality rate up to 100% in the absence of any intervention. Therefore, it is extremely important to diagnose these cases at an early stage. The objectives of this study were to assess the diagnostic performance of computed tomography (CT) and diffusion-weighted MR imaging (DW-MRI) in the diagnosis of infection in pancreatic collections.

Materials and methods: Prospective observational study of abdominal collections due to pancreatitis that underwent both CT and DW-MRI from August 2018 to July 2020 were enrolled in the study. The collections were analysed for infections - air foci in CT and diffusion restriction on DW-MRI.

Results: Of the 39 patients recruited in the study, infected collections were present in 17, and 22 cases had sterile collections. On CT, air foci within the collection were present only in seven of the cases in our study (sensitivity 35%, specificity 95.4%, PPV 85.7%, NPV 65.6%). DW-MRI detected infection in all 17 cases (sensitivity 100%, specificity 72.7%, PPV 74%, NPV 100%).

Discussion: Thirteen out of 17 collections suspicious for infection on DW-MR showed microbiological growth on culture examination. We believe that this is likely due to the patient's prior antibiotic use, which might have altered the micro-environment or inflammatory cell and bacterial content of the collection.

Conclusion: DW-MRI is complementary and superior to CT in detecting infection in pancreatic collections. CT may be used to detect disease burden, extent and vascular complication.

## Introduction

Pancreatitis in its acute inflammatory form may manifest with symptoms ranging from mild epigastric pain to life-threatening multiple organ failure. In its chronic state, it may present with mild abdominal pain, indigestion, weight loss, and steatorrhea. According to the Revised Atlanta classification, acute pancreatitis can be divided into interstitial pancreatitis (80%) and necrotizing pancreatitis (5%-15%) [1]. Interstitial oedematous pancreatitis may be associated with acute pancreatic fluid collection (APFC), which, if not resolved, may, over time, progress to form pancreatic pseudocyst. Necrotizing pancreatitis may be related to acute necrotic collection (ANC) and, over time, with walled-off necrosis (WON). These collections can be sterile or infected [1].

Severe necrotizing pancreatitis is associated with high mortality rates of 30% to 40% [2]. The majority of the death in the initial phase of this disease is due to multiple organ failure; however, the local and systemic infectious complications play a significant role in the mortality in the later phase [3]. The mortality associated with necrotizing pancreatitis is less than 10% if the necrotic tissue remains sterile [4-7]. But once the infection of the pancreatic necrosis sets in, the mortality could be high as 100% if left untreated, which could be lowered only to 15% to 50% even after the surgical intervention [8,9]. A multimodality approach is ideal in managing acute pancreatitis and its complications in which the diagnosis of infection plays a crucial role. Hence, early and prompt diagnosis plays a vital role in immediate treatment and prevention of complications.

Clinical symptomatology such as fever is not reliable as indicators of infection in acute pancreatitis [10]. Various clinical and biochemical parameters (hypotension, APACHE II score at 24 hours of hospital admission, Procalcitonin [PCT], Interleukin-8 [IL-8], blood urea nitrogen) can predict early infection in AP [11-14]. The non-specificity of these biochemical tests precludes their use in routine practice.

Ultrasound, which is used as the first modality of radiological investigation in acute pancreatitis, has failed to identify necrosis and has a limited role in characterizing fluid collections. The main problem is that the pancreas may not be visible in all patients because of the gaseous abdomen. PET CT with 18F-FDG-labelled autologous leukocytes has good diagnostic performance [15]. However, the exposure to ionizing radiation, risk of hypersensitivity reactions to contrast media, inability to use in pregnancy and in renal impairment have raised the need for a better diagnostic approach for early and accurate diagnosis of infection in pancreatic collections.

CECT Abdomen (Pancreatic protocol) remains the most common investigation done for diagnosis and evaluation of acute pancreatitis. MRI Abdomen has an emerging role in the diagnosis of the pancreatic collection as well as infection in the setting of acute pancreatitis [16,17].

The objectives of this study were to assess the diagnostic performance of CECT in the diagnosis of infection in the pancreatic collection, assess the diagnostic performance of diffusion-weighted MR imaging (DW-MRI) in the diagnosis of infection in the pancreatic collection and compare the diagnostic performance of CECT and DW-MRI in the diagnosis of infection in pancreatic collections.

## Materials and methods

Patients

**Figure 1:**
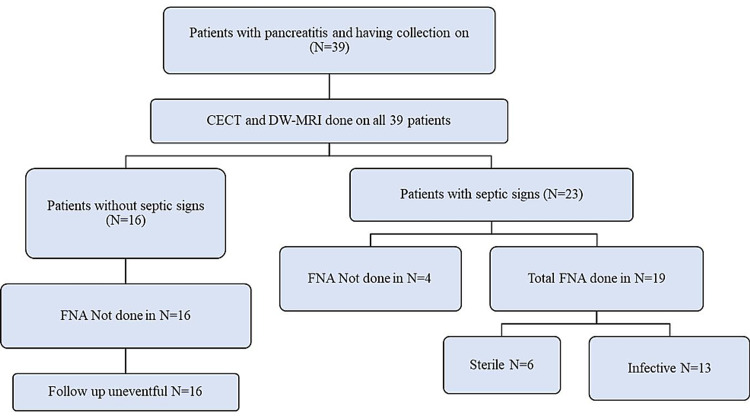
Flow of patients through the study.

This prospective single-group observational study was carried out after approval from the Institutional Research Committee and Institutional Ethics committee (AIIMS/IEC/2018/589). From August 2018 to July 2020, all cases, those who were diagnosed with acute pancreatitis on clinical and had a pancreatic or peripancreatic fluid collection on USG were enrolled in the study. The exclusion criteria were general MRI contraindications and history of intervention for drainage of the collection in the past month. A total of 39 patients were enrolled in this study as shown in Figure 1.

Imaging technique

All abdominal CT scans were performed on Siemens Somatom Definition Flash Dual Energy 128*2 Slice Multi-Detector CT scanner after injection of 100 mL of iodinated contrast material. Scans were acquired in the pancreatic and hepatic venous phases.

All MRI examinations were performed on GE Discovery 750W 3-T (Fairfield, CT, USA) with 32-channel phased-array body coil. Our protocol included axial T2‑weighted non-fat saturated fast spin-echo sequence, coronal T2 scans, T1‑weighted three‑dimensional gradient‑echo sequence with fat suppression and an axial respiratory‑triggered diffusion‑weighted single‑shot echo‑planar sequence with fat suppression.

Image analysis

Two radiologists (with over 12 years and two years of experience) interpreted the patient's CECT and MRI. The findings were recorded only after the two radiologists reached a mutual consensus. CECT images were analysed for collections according to the Revised Atlanta classification. MCTSI was given in all of the cases. The presence of the air bubbles in the collections was considered to be an imaging parameter for infection as done routinely. All MCTSI values were measured directly on the workstation console (syngo via VA 30; Siemens) by image analysis. For MRI, the two readers analysed pancreatic collections for the presence or absence of diffusion restriction. (Presence: persistence of DWI high signal on b-value 1,000 s/mm^2^ images compared to images at b-value 50 s/mm^2^ and Absence: no persistence of DWI high signal on b-value 1000 s/mm^2^ images compared to images at b-value 50 s/mm^2^).

Fine-needle aspiration procedure

All drainage was carried out within three days of MRI, either under ultrasound or CT guidance, with most of them having FNA on the same date of imaging. Percutaneous access was achieved using an 18‑gauge puncture needle followed by pigtail drainage if needed. 5-20 mL of the fluid was separately collected and transferred into a sterile universal container. The collection was considered sterile if the culture yields no growth even after 48 hours of incubation.

Statistical analysis

The data were evaluated using Statistical Package for Social Sciences (SPSS Inc., Chicago, IL, USA) version 23. Qualitative variables like gender, etiological factors, and type of peripancreatic collection were described using frequency and percentages. Diagnostic performance of CECT and qualitative DW-MRI in non-invasive detection of infection in pancreatic/peripancreatic collection was done considering FNA culture analysis and clinical follow-up as outcome reference, the sensitivity, specificity, PPV, and NPV were calculated for both the investigation.

## Results

Of the 39 patients recruited in the study, 17 were infected and 22 were sterile collections. On CT, the air within the collection was present only in seven cases. The distributions of the various pancreatic/peripancreatic fluid collection studied in the study using CECT were 21 ANCs, 13 WONs and five APFCs (Table 1). The mean modified CT severity index of the sterile group was calculated as 7.36+1.67 and that of the infected group was 8.58+1.69.

**Table 1:** Types of collections on CT and MRI with modified CT severity index score.

Collections	CECT		DW-MRI		Outcome reference		MCTSI (Mean)
	Infected	Sterile	Infected	Sterile	Culture	Follow-up	
Infected (n=17)	06	11	17	00	13	04	8.58+1.69
Sterile (n=22)	01	21	06	16	06	16	7.36+1.67

Six out of seven collections that were pre-diagnosed to be infected based on the presence of air on CECT were found to be infected on culture analysis and follow-up. The remaining one collection did not grow any microbial growth even after 48 hours of aerobic microbial culture. Amongst other 32 collections which were classified as sterile based on the absence of gas bubbles on CECT, 11 were found to be infected on culture analysis and follow up and 21 were sterile on the basis of outcome reference. CECT showed a sensitivity of 35.2% (6/17), a specificity of 95.4% (21/22), a positive predictive value of 85.7% (6/7), and a negative predictive value of 65.6% (21/32).

**Figure 2:**
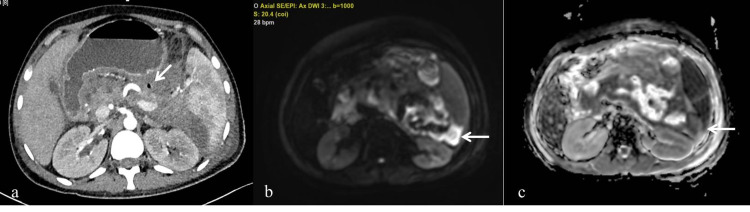
A 26-year-old-male, presented with alcohol-induced acute pancreatitis, fine-needle aspiration analysis yield infective collection with Escherichia coli growth on microbial culture. (a) Axial contrast CT pancreatic phase showing pancreatic parenchymal destruction with collection in the lesser sac, anterior pararenal space and air foci within (arrow). (b, c) Diffusion-weighted MR image at b-value of 1,000 s/mm^2^ and corresponding ADC map showing diffusion restriction in the collection (arrow).

**Figure 3:**
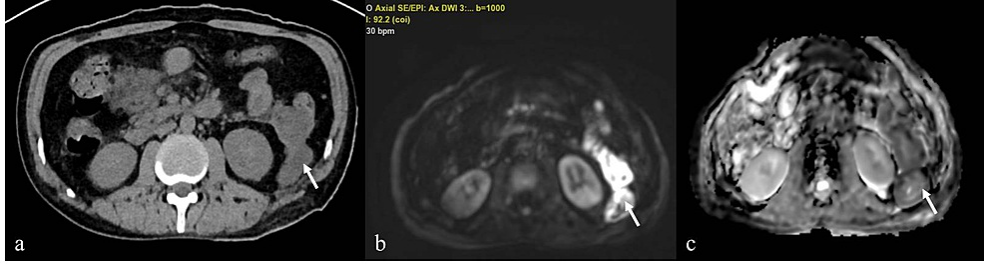
A 33-year-old-male presented with alcohol-induced acute pancreatitis. (a) Axial non-contrast CT shows heterogeneous collection along the left paracolic gutter without air (arrow). (b, c) Diffusion-weighted MR images and corresponding ADC map showing diffusion restriction in the collection (arrow). He had infected collection confirmed by fine-needle aspiration.

DW-MRI showed diffusion restriction in all 17 infected collections (sensitivity 100%, NPV 100%) (Figures 2a-2c,3a-3c).

In addition, diffusion restriction was seen in six collections which did not show any microbiological growth (specificity 72%, PPV 74%). 16 collections that were sterile did not show diffusion restriction on MRI. The diagnostic performance of CT and DW-MRI is shown in Table 2.

**Table 2:** Diagnostic performance of computed tomography and diffusion-weighted MRI.

CECT	Outcome		Sensitivity	Specificity	PPV	NPV
	Infected	Sterile				
Infected (Air foci)	06	01	35.2%	95.4%	85.7%	65.6%
Sterile	11	21				
DW-MRI						
Infected	17	06	100%	72.7%	73.9%	100%
Sterile	00	16				

## Discussion

Pancreatitis is evolving as a life-threatening disease; early diagnosis and assessment of necrosis and infection play a vital role in preventing complications and disease management. However, no single best method has been described in the literature to assess disease severity and early diagnosis. Identification of a perfect radiological tool plays an essential role in judging disease diagnosis and severity. Therefore, the aim of the present study was to evaluate the role of CECT and MRI in assessing infection in pancreatic collections.

The common diagnostic investigation performed for the detection and assessment of PFC is contrast-enhanced computed tomography (CT). Suspicion of infection at CECT is based on the findings of gas bubbles in the collection. Thirty-five percent of the infected collections were detected by CT. In the early stages of acute pancreatitis, most infected collections do not demonstrate air bubbles on CECT. In fact, in our study, 65% of the infected collections had no air bubbles. In such cases where the infection is suspected clinically, FNA sampling from the collection should be performed to diagnose infection. DW‑MRI is a novel technique. It has now been well established as a sequence for diagnosing various non-infective and infective intracranial pathologies such as subdural empyema and brain abscesses. The diagnostic use of the DW‑MRI sequence for various intra-abdominal pathologies is also increasing nowadays. Areas of diffusion restriction give dark pixels on ADC images. On the other hand, areas of fast diffusion show bright pixels on the ADC image.

Qualitative MRI can detect infection within AFCs with the sensitivity and NPV of 100%, PPV of 73.9%, and specificity of 73.9%. This finding complies with the study done by Islim et al. [16] in which the sensitivity and NPV of 100%, PPV of 90.9%, and specificity of ~90.9%. It also complies with the study done by Borens et al. [17] which had a sensitivity of 75%, a specificity of 96%, NPV of 90%, and PPV of 90%.

In our study, diffusion restriction at the centre of the content was seen in eight of the 39 patients, whereas peripheral diffusion restriction was seen in 21 patients. Usually, the abdominopelvic and hepatic abscesses show high signals in central portions on DW-MRI [18,19]. This is likely due to the high pancreatic enzyme level in the pancreatic collection compared to a hepatic and abdominopelvic abscess, which breaks down the inflammatory cells, resulting in reduced cellularity. This, in turn, causes a decrease in diffusion hyperintensity in central portion in pancreatic infected collections. Thus, we suggest considering either the content or peripheral diffusion restriction as a sign of infection.

It is to note that, in our study, the cultural results were sterile in six of the patients in which septic symptoms and signs were present. Septic symptoms and signs were probably associated with systemic inflammatory response syndrome (SIRS). Of all the 39 patients, the microbial culture examination was done for 19/23 of the diffusion restricting samples and 3/16 of the non-diffusion restricting samples. Microbiological culture results were in accordance with the DWI results in almost all of them except in three of the patients. In these cases, the positive DW-MRI results were in disagreement with the culture results. We believe that this is likely due to the patient's prior antibiotic use, which might have altered the microbiological flora and content of the collection.

The study has a few limitations. Due to ethical reasons and the possibility of introducing secondary infection, we could not do the microbial examination in 20 patients (four with septic symptoms and 16 without septic symptoms). However, it is to note that this did not delay the treatment if an infection was suspected clinically. In our study only aerobic microbial analysis is done as previous studies have shown that the most common infecting organisms in the peripancreatic collection are aerobic [4,20-24]. We did not include anaerobic and fungal culture analysis for fine needle aspirate. The present study is a continuation of our previously published pilot study [24]. Our previous study had less sample size (seven infected; 11 sterile collections) and used quantitative parameters. We could not generalize our findings in this subgroup. But with increasing experience and more sample size, we recommend the use of DW-MRI in the early detection of infected collections.

## Conclusions

In conclusion, the low sensitivity of CECT with additional radiation exposure compromised its daily practice usage. We attempted to apply the DWI MR at early non-invasive detection of infection in the pancreatic and peripancreatic collection in the setting of acute pancreatitis. Diffusion restriction in either the centre or the periphery of the pancreatic collections is an indicator of early infection. So, we recommend using CECT for evaluation of complications and extent of disease while DW-MRI should be additionally used to detect early infection in pancreatic collections.
